# Pyelo-Nephritis Consequent upon the Presence of Calculi and Over-distention of the Bladder for Three Weeks—Twenty-one Calculi Found in the Bladder

**Published:** 1883-11

**Authors:** A. R. Davidson

**Affiliations:** Professor of Medical Chemistry and Toxicology in Niagara University


### PYELO-NEPHRITIS CONSEQUENT UPON THE PRESENCE OF CALCULI AND OVER-DISTENTION OF THE BLADDER FOR THREE WEEKS—TWENTY-ONE CALCULI FOUND IN THE BLADDER.

REPORTED BY A. R. DAVIDSON, M. D.,
Professor of Medical Chemistry and Toxicology in Niagara University.

On the evening of September 23, 1883, I was called to attend C. J. F., age 58. The history of the case, as far as could be obtained, was as follows: For some years he had occasionally suffered with frequent desire to micturate, and some pain in the bladder, but these attacks were only occasional, and attributed by him to "catching cold." Three weeks previous to my first visit he had spent a week in Michigan, and upon his return had two chills, followed by fever which his physician pronounced to be malarial. Increased frequency of micturition followed (every half hour) and a continued fever, his strength failed rapidly, so that he was confined to bed, and according to his statement was treated assiduously for disease of the liver and lungs. Diabetes, it is said, was also suspected, on account of the large quantity of urine voided, it having become necessary for him, during the last four days, to arise every fifteen or twenty minutes to pass water, though the quantity passed was less than an ounce at each attempt. His physician was a Homœopath in large practice. Upon the day on which I was called to the case, a consultation had been held with a brother practitioner, and an attempt made to pass a catheter. After an hour's effort, it was stated that one instrument had been introduced into the bladder, but no urine obtained. The poor fellow asserted that he had suffered untold tortures, and that he did not want any more attempts made to pass catheters upon him. An examination showed an apparently normal liver and healthy lungs, and also, as was expected, a large ovoid tumor in the abdomen, reaching nearly to the umbilicus. A finger introduced into the rectum showed, also, the presence of a very large and painful prostate. There was also a contracted meatus urinarius. At the urgent request of the patient, and fearing that some violence might have been used in previous attempts, the passage of a catheter was deferred until the following morning. Some of the urine was obtained, and an analysis in my laboratory yielded the following result:

Color, very pale, No. 1 Vogel.
Odor, ammoniacal.
Reaction, alkaline (from ammonia).
Specific gravity, 1.006.
Deposit, a little white, flocculent precipitate, easily diffused.
Urea, 1.6 grains to the ounce.
Albumen, about one-tenth of one per cent.
Sugar absent.

MICROSCOPICAL EXAMINATION.

Crystals of triple phosphate, much bladder epithelium, pus small in quantity, but many corpuscles having branched or tooth-like projections. Kidney epithelium very sparingly present, but much epithelial detritus.

The examination indicated that pyelitis existed, and, probably, from the very low proportion of urea, a suppurative, interstitial nephritis. Recognizing, therefore, the gravity of the case, I insisted upon a consultation, and Dr. Tremaine, U. S. A., was called in, and during the further progress of the case the patient had the advantage of his able counsel.

On the morning of the 24th I introduced a French Coodéé Catheter, No. 17, F., without difficulty, although the prostatic portion of the urethra was extremely sensitive. Three pints of ammoniacal urine was obtained. As the patient then complained of some pain, the catheter was withdrawn, leaving still a good deal of water in the bladder.

On the following morning the bladder was emptied. Such great pain followed, located by the patient in the glans penis, that I felt certain that a stone was present in the bladder. The contraction of the bladder on the enlarged prostate would not account for the severity of the pain. My inquiries for the ordinary symptoms of the presence of a calculus before he was taken sick were answered in the negative. Especially, he assured me that he has suffered no pain after making water, but rather before.*

In view of his great debility and the diseased condition of the kidneys, any operation was out of the question at the time, and therefore nothing was to be gained by an exploration of the bladder. It was thought, however, that a stone was detected by subsequent catheterization with a soft instrument. The urine was not again entirely withdrawn, and the patient, though very weak, was absolutely free from pain. Palpation over the region of the kidney caused some pain, notably on the left side. The urine became less ammoniacal, and polyuria ceased, but the amount of urea excreted did not increase. The tongue grew slowly more red, dry and contracted, the powers of life rapidly failed, the senses became impaired and he died October 1st.

The family of the deceased being people of intelligence and culture, my urgent request for a post mortem was granted. The result was interesting and instructive.

*Kidneys.* On cutting the ureters pus dropped freely from both. On section much pus was found in both pelves. In the pelvis of the left kidney were five phosphatic calculi, the largest the size of a white bean, their total weight being 0.900 grammes.

*Bladder.* On grasping the bladder the sensation was that of taking hold of a bag of marbles. Twenty-one calculi were

* The absence of this important symptom was undoubtedly due to the considerable amount of "residual urine" habitually present in the bladder.

found within, varying in size from a small hen's egg to a hickory nut; their total weight was 139.9 grammes. One of them sawed through showed a nucleus of uric acid; more than three-fourths of its diameter, externally, was composed of a uniform layer of phosphates, showing that there had been an uninterrupted deposit of triple phosphates, and this later growth was probably acquired during the time that his bladder was left filled with ammoniacal urine in his last sickness.

The coats of the bladder were thickened, the blood vessels dilated, but the mucus surface did not present the appearance of inflammation to any marked degree. Both lobes of the prostate gland were found to be enlarged, the median portion markedly so, and a small nipple-like projection upon it just filling the internal orifice of the urethra. The case is of peculiar interest, first in the large number of calculi of considerable size found in the bladder, and secondly in the indication of their very rapid growth. This is not so remarkable when we consider that the urine contains from two to four grains of phosphates in each ounce, and the large amount of it which was constantly present in the bladder. When urine is rendered alkaline by the formation of carbonate of ammonia, resulting from the decomposition of urea, not only is the phosphate of calcium precipitated (being soluble only in acid fluid, and not in alkaline ones), but it also forms, by the action of the ammonia on the phosphate of magnesia, always present in the urine, a triple phosphate of ammonio-magnesia phosphate. The case is different when the alkaline condition ot the urine does not depend upon carbonate of ammonia, but on carbonate of potassium, sodium, or some other fixed alkali. Then no triple phosphate can form, and the sediment will consist only of phosphate of calcium. The calculi in the kidney were composed of large crystals of triple phosphates, loosely aggregated, so that they easily crumbled and were undoubtedly formed during the patient's last sickness. A recognition of the condition of the bladder two or three weeks previous to my first visit, with proper treatment of the case, would probably have prevented any involvement of the kidney.

